# MKK7 deficiency in mature neurons impairs parental behavior in mice

**DOI:** 10.1111/gtc.12816

**Published:** 2020-11-18

**Authors:** Tadashi Shin, Yuichi Hiraoka, Tokiwa Yamasaki, Jamey D. Marth, Josef M. Penninger, Masami Kanai‐Azuma, Kohichi Tanaka, Satoshi Kofuji, Hiroshi Nishina

**Affiliations:** ^1^ Department of Developmental and Regenerative Biology Medical Research Institute Tokyo Medical and Dental University (TMDU) Tokyo Japan; ^2^ Department of Molecular Neuroscience Medical Research Institute Tokyo Medical and Dental University (TMDU) Tokyo Japan; ^3^ Department of Physiology Keio University School of Medicine Tokyo Japan; ^4^ Center for Nanomedicine Department of Molecular, Cellular and Developmental Biology SBP Medical Discovery Institute University of California Santa Barbara Santa Barbara CA USA; ^5^ IMBA Institute of Molecular Biotechnology of the Austrian Academy of Sciences Vienna Austria; ^6^ Department of Medical Genetics Life Sciences Institute University of British Columbia Vancouver BC Canada; ^7^ Department of Experimental Animal Model for Human Disease Center for Experimental Animals Tokyo Medical and Dental University (TMDU) Tokyo Japan

**Keywords:** JNK, mature neuron, MKK7, parental behavior

## Abstract

c‐Jun N‐terminal kinases (JNKs) are constitutively activated in mammalian brains and are indispensable for their development and neural functions. MKK7 is an upstream activator of all JNKs. However, whether the common JNK signaling pathway regulates the brain's control of social behavior remains unclear. Here, we show that female mice in which *Mkk7* is deleted specifically in mature neurons (*Mkk7^flox/flox^Syn‐Cre* mice) give birth to a normal number of pups but fail to raise them due to a defect in pup retrieval. To explore the mechanism underlying this abnormality, we performed comprehensive behavioral tests. *Mkk7^flox/flox^Syn‐Cre* mice showed normal locomotor functions and cognitive ability but exhibited depression‐like behavior. cDNA microarray analysis of mutant brain revealed an altered gene expression pattern. Quantitative RT‐PCR analysis demonstrated that mRNA expression levels of genes related to neural signaling pathways and a calcium channel were significantly different from controls. In addition, loss of neural MKK7 had unexpected regulatory effects on gene expression patterns in oligodendrocytes. These findings indicate that MKK7 has an important role in regulating the gene expression patterns responsible for promoting normal social behavior and staving off depression.

## Graphical abstract

c‐Jun N‐terminal kinase (JNK) signaling has well established, important roles in mammalian brain development and neural functions. However, whether the mitogen‐activated protein kinase kinase 7 (MKK7), a specific upstream activator of JNK, regulates social behavior remains unclear. In this study, we demonstrate that the MKK7‐JNK pathway in mature neurons regulates mouse mental status and plays a critical role in parental behavior.

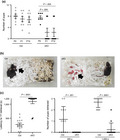

## 1 INTRODUCTION

The adult mammalian brain is a highly complex organ that controls key physiological functions such as locomotion, cognition and mental activity (DiCarlo et al., 2012; Hultborn & Nielsen, 2007; Ito, 2008). The brain is composed of various cell types, including neural stem/progenitor cells, immature and mature neurons and glial cells (Allen & Lyons, 2018; Zeng & Sanes, 2017). Interaction between neurons and glial cells mutually regulates their differentiation and maturation (Gibson et al., 2014; Shaham, 2005).

Social behavior is the name given to the interactions that occur among individuals and that help them to live together more peaceably. Parental behavior is a subtype of social behavior and is essential for the survival and proper mental and physical development of mammalian offspring (Dulac et al., 2014). In rodents, parental behavior includes nest building and pup retrieval (Kuroda et al., 2011), activities that are governed by maternal locomotor activity, cognitive ability and mental status (Paquin et al., 2020; Zhang et al., 2020). In humans, avoidance of mental illness is particularly socially important, with postpartum depression being one of the well‐known risk factors for child abuse (Choi et al., 2010).

In most mammalian organs, c‐Jun N‐terminal kinases (JNKs) are activated in response to an external stimulus and regulate a variety of stress responses (Johnson & Nakamura, 2007). In contrast, as we have previously reported (Yamasaki et al., 2017), JNKs are constitutively activated in mammalian brain and are indispensable for its development and neural functions (Coffey, 2014). In most organs, JNKs are activated by either mitogen‐activated protein kinase kinase 4 (MKK4) or MKK7 upon receipt of a stress stimulus. In mouse brain, Nestin‐Cre‐mediated deletion of *Mkk4* or *Mkk7* causes developmental defects, indicating that MKK4/7‐JNK signaling is necessary not only for stress responses but also for early development of the brain (Wang et al., 2007; Yamasaki et al., 2011). Similarly, Syn‐Cre‐dependent deletion of *Mkk7*, which is neuron‐specific, impairs circadian rhythms in 10‐week‐old mice, with 8‐month‐old mutants showing an age‐dependent decrease in locomotor activity (Yamasaki et al., 2017). Thus, the MKK7‐JNK pathway regulates diverse neural functions in addition to brain development. However, whether the MKK7‐JNK pathway participates in the social behaviors initiated by the brain is not known.

In this study, we investigated MKK7 functions in adult mouse brain using animals lacking *Mkk7* specifically in mature neurons. We report an important link between MKK7‐JNK signaling and the parental behavior of pup nurturing.

## 2 RESULTS

### 2.1 *Mkk7^flox/flox^Syn‐Cre* female mice exhibit impaired parental behavior

**Figure 1:**
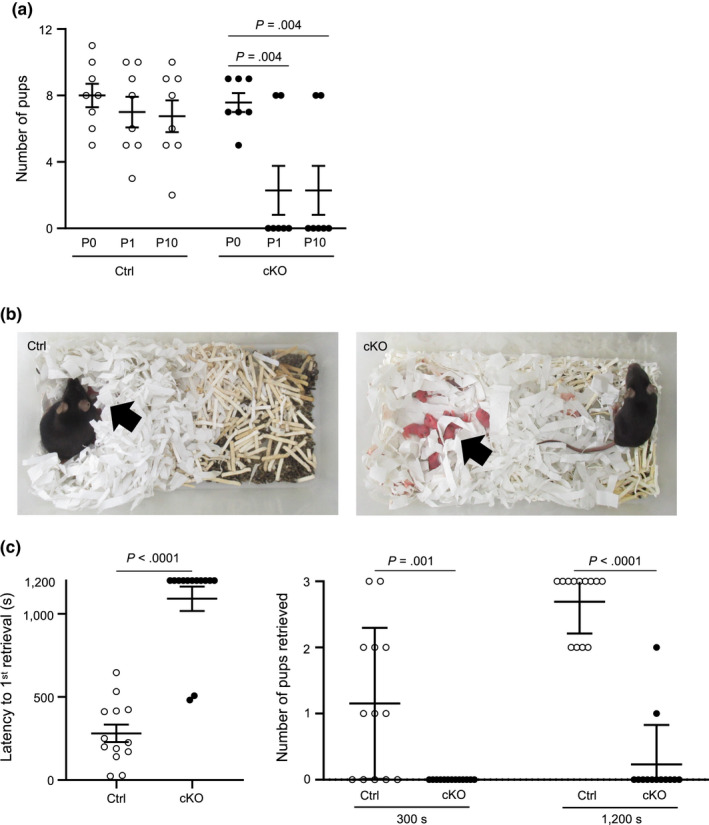
Impaired pup retrieval in*Mkk7^flox/flox^Syn‐Cre*mice. (a) Number of surviving pups in single litters born of 14‐ to 21‐week‐old control (Ctrl) (*n* = 8) or cKO (*n* = 7) female mice at P0, P1 and P10. (b) Representative images of nest building for P0 pups (a nurturing behavior) by an 18‐week‐old control female mouse (left) and a 16‐week‐old cKO female mouse (right). (c) Pup retrieval assays. Left: Time to retrieval of the first of three pups by 14‐ to 18‐week‐old virgin control and cKO female mice*(n* = 13). Right: Numbers of pups retrieved by 300 s and 1,200 s by the mice in the left panel. For (a) and (c), data are the means ± *SEM*

Previously, we reported that neuron‐specific *Mkk7*‐deficient mice (*Mkk7^flox/flox^Syn‐Cre* mice, hereafter designated cKO mice) were born at the expected Mendelian ratio and were fertile (Yamasaki et al., 2017). During our regular maintenance of these animals, however, we noted that we rarely obtained pups from cKO female mice. To assess this observation quantitatively, we compared the number of pups surviving until postnatal day (P) 0, P1 and P10 between control and cKO female mice. Although there was no difference in the number of pups born, very few offspring of cKO females survived even until P1 (Figure 1a). Because cKO females had normal mammary glands, we hypothesized that cKO females might have a defect in parental behavior.

To test this theory, we first evaluated nest building. We found that cKO female mice were not able to build their nests as effectively as control mice (Figure 1b). Secondly, we performed pup retrieval assays using virgin control and cKO female mice (to exclude confounding factors such as hormonal milieu and physical stress induced by parturition (Kuroda et al., 2011)). Most control females successfully retrieved a set of newborn pups and brought them to the nest within 1,200 s (Figure 1c and Video S1). In contrast, almost all cKO females were indifferent to the newborn pups and did not bring them to the nest (Figure 1c and Video S2). These observations are strong evidence that cKO female mice have a defect in parental behavior.

### 2.2 *Mkk7^flox/flox^syn‐cre* male and female mice show depression‐like behavioR

**Figure 2:**
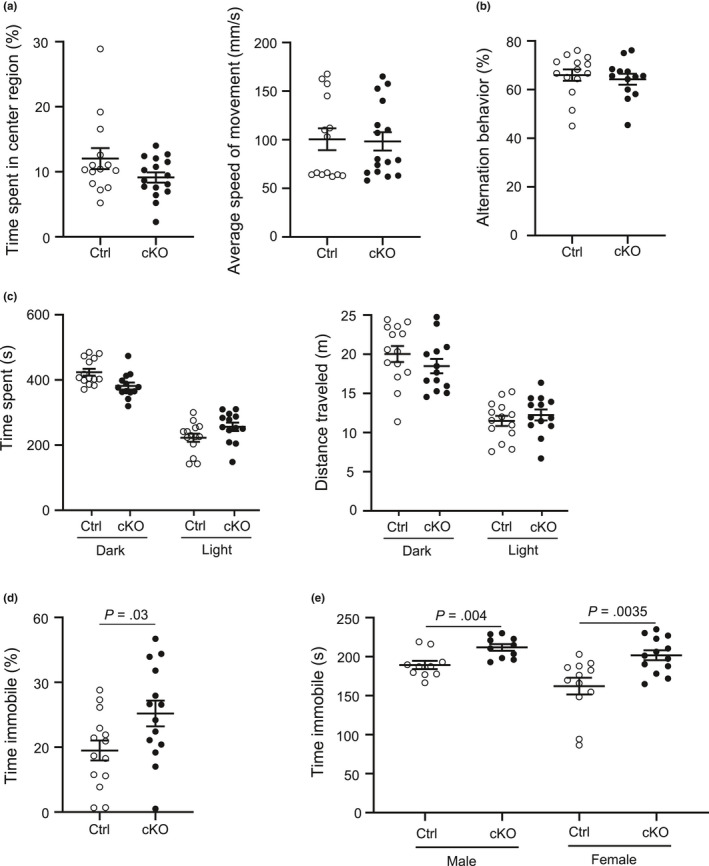
Depression‐like behaviors of*Mkk7^flox/flox^Syn‐Cre*mice. (a) Open field test. Left: Percentage of time spent in the center of a container by control (*n* = 14) and cKO (*n* = 16) mice at 12–16 weeks of age. Right: Average speed of movement (mm/s) of the mice in the left panel. (b) Y‐maze test. Percentage of alternation behaviors by control (*n* = 13) and cKO (*n* = 14) mice at 12–16 weeks of age. (c) Light–dark test. Left. Time spent in the dark or light chambers by control (*n* = 14) and cKO (*n* = 13) mice at 12–16 weeks of age. Right: Distance travelled into the light chamber by the mice in the left panel. (d) Tail suspension test. Percentage of time of immobility measured over 2–4 min of 12‐ to 16‐week‐old control and cKO mice (*n* = 14) suspended by their tails. (e) Forced swim test. Percentage of time of immobility measured over 2–4 min of 12‐ to 16‐week‐old control male (*n* = 12), cKO male (*n* = 13), control female (*n* = 10) and cKO female (*n* = 10) mice immersed in a water‐filled cylinder. For all panels, data are the mean ± *SEM*

To explore the causes of the impaired parental behavior of cKO female mice, we tested the locomotor activity, cognitive ability and mental status of cKO males and females. The open field test revealed no abnormalities in the locomotor activity of cKO mice (Figure 2a). Similarly, the Y‐maze test showed that cKO mice have normal cognitive ability (Figure 2b). In the light–dark box test, which is a test of mental status, cKO and control mice showed comparable levels of anxiety‐related behavior (Figure 2c). On the other hand, compared to controls, cKO mice showed significantly longer periods of immobility in the tail suspension test (Figure 2d) and the forced swim test (Figure 2e), indicating depression. Taken together, these data indicate that cKO mice suffer from depression‐like behavior but have normal locomotor activity and cognitive ability.

### 2.3 *Mkk7* is deleted in mature but not immature neurons in *Mkk7^flox/flox^syn‐cre* mice

**Figure 3:**
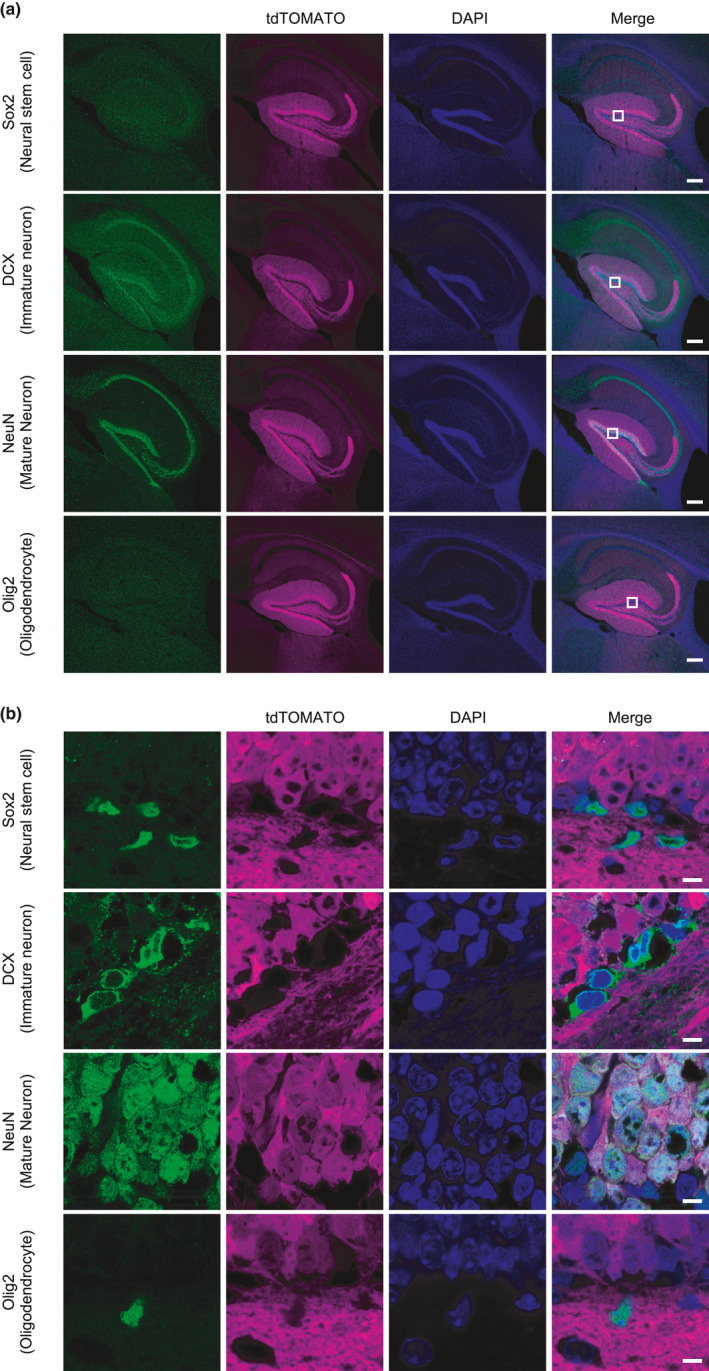
Expression of*Syn‐Cre*in mature neurons. (a) Representative Hippocampal sections (40 µm) of 8‐week‐old*tdTomato^flox/+^Syn‐Cre*mice that were stained with primary Abs recognizing Sox2 (neural stem cell marker), DCX (immature neuron), NeuN (mature neuron) or Olig2 (oligodendrocyte). Red, tdTomato; green, AlexaFluor 488‐labeled secondary Abs. Blue, Hoechst 33342 (nucleus). Scale bars, 200 µm. (b) High magnification images of the areas in the white squares in (a). Scale bars, 5 µm

To pinpoint the Mkk7‐deficient cells responsible for the parental behavior defect in cKO mice, we performed immunohistochemical analyses of *tdTomato^flox/+^Syn‐Cre* reporter mice, in which tdTomato is expressed in cells in which Syn‐Cre is functioning (Madisen et al., 2010). We examined tdTomato expression in the hippocampal subgranular zones (SGZs) of the brain because these areas are enriched for neural stem cells, immature neurons and mature neurons (Ming & Song, 2011). We found that tdTomato was not co‐expressed with either Sox2, a neural stem cell marker, doublecortin (DCX), an immature neuron marker, or Olig2, an oligodendrocyte marker. Instead, tdTomato was strongly co‐expressed in SGZs with NeuN, a mature neuron marker (Figure 3). These data indicate that Syn‐Cre recombinase is exclusively expressed in mature neurons in adult mouse brain, and that the phenotypes of cKO mice are caused by deletion of *Mkk7* specifically in mature neurons.

### 2.4 Gene expression profiles are altered in *Mkk7^flox/flox^syn‐cre* mice

**Figure 4:**
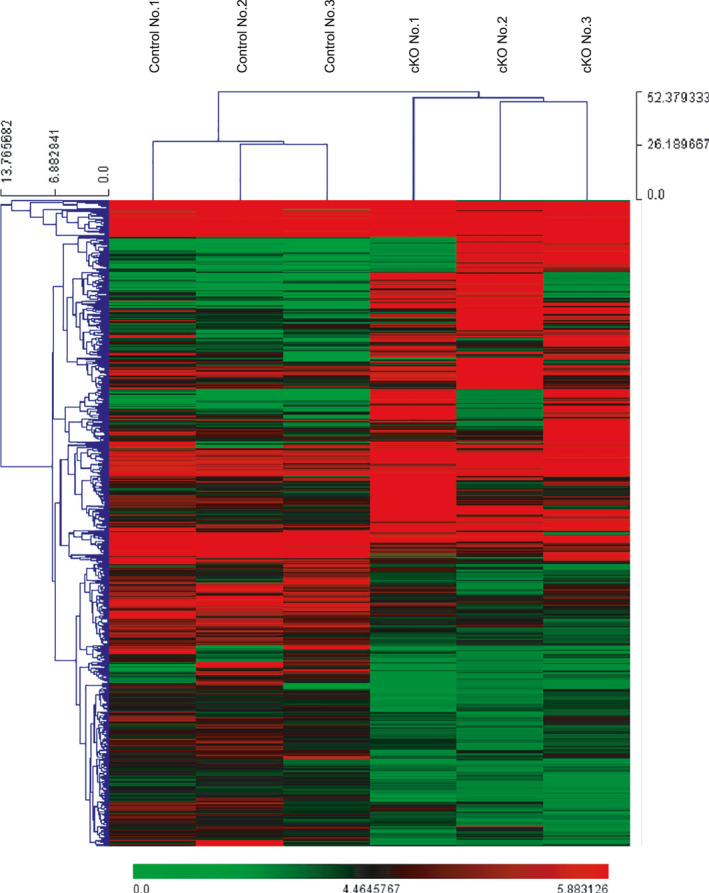
Altered gene expression patterns in*Mkk7^flox/flox^Syn‐Cre*mouse brains. Heatmap showing supervised hierarchical clustering of 4,839 probes found to be differentially expressed in cKO mice (*n* = 3) compared with control mice (*n* = 3) at 8 weeks of age. The relative mRNA level of a given gene is indicated as either high (red) or low (green)

To more closely examine the effects of *Mkk7* deficiency on mature neurons, we analyzed the brains of young (8–10 weeks old) cKO mice, a time before their brain functions would be influenced by environmental cues. We first examined the phosphorylation status of JNK and one of its downstream targets, the transcription factor c‐Jun. Consistent with our previous report (Yamasaki et al., 2017), levels of phosphorylated JNK and c‐Jun were decreased in the brains of cKO mice (Figure S1). Furthermore, cDNA microarray analysis of cKO mouse brains at 8 weeks of age identified 4,839 probes showing significant differences (*p* < .05). We then subjected these data to unsupervised cluster analysis and supervised hierarchical cluster analysis (Figure 4). The unsupervised cluster analysis clearly divided the data sets into the control and cKO groups, indicating that our results faithfully reflected the effects of *Mkk7* deletion. The supervised hierarchical cluster analysis revealed that the expression profile of the cKO group could be further divided into genes that were upregulated and those that were downregulated compared to controls. After considering probes for the same target genes, we identified 35 genes that were more than 2‐fold upregulated in cKO mouse brains over controls, and 112 genes that were more than 2‐fold downregulated (Tables S1 and S2). Examination of the nature of these genes confirmed that MKK7 regulates gene expression patterns essential for neural functions.

### 2.5 Expression patterns of neuron‐related and oligodendrocyte‐related genes are altered in *mkk7^flox/flox^syn‐cre* mice

**Figure 5:**
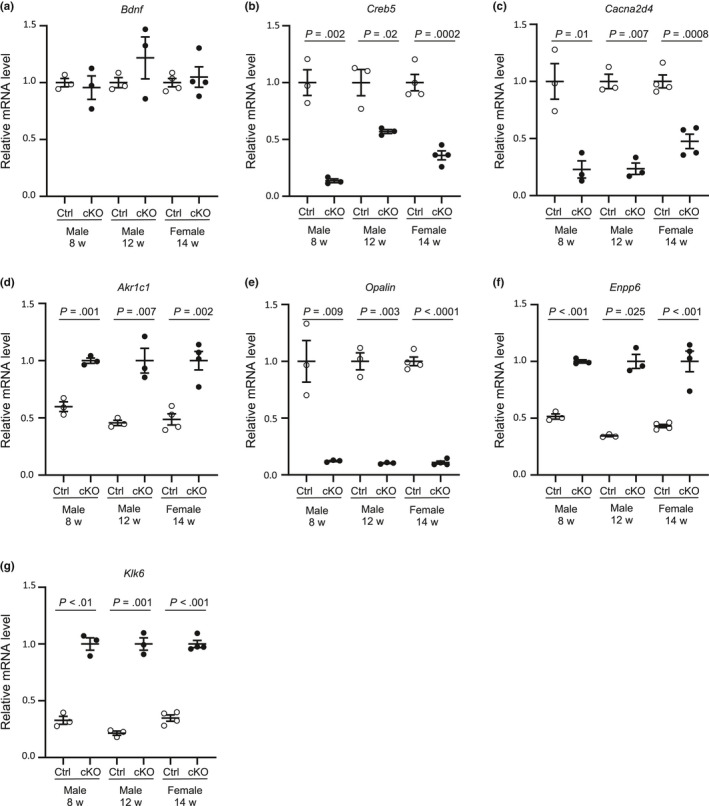
mRNA levels of genes related to neural functions and oligodendrocyte maturation in*Mkk7^flox/flox^Syn‐Cre*brains. Quantitative RT‐PCR analyses were performed to detect mRNAs for: (a)*Bdnf*, (b)*Creb5*, (c)*Cacna2d4*, (d)*Akr1c1*, (e)*Opalin*, (f)*Enpp6*and (g)*Klk6*in brain tissues of 8‐ or 12‐week‐old control and cKO male mice (*n* = 3/group) and 14‐week‐old control and cKO female mice (*n* = 4/group). mRNA levels are expressed relative to*Gapdh*.Data are the mean ± *SEM*

We next examined changes in gene expression in cKO brains by qRT‐PCR. mRNA levels of brain‐derived neurotrophic factor (BDNF), which is essential for neural activities, were not changed in male and female cKO whole brain tissues (Figure 5a). On the other hand, mRNA levels of genes related to neural signaling pathways such as *Creb5* (cAMP‐responsive element binding protein‐5) and *Cacna2d4* mRNA encoding a calcium channel (calcium voltage‐gated channel auxiliary subunit alpha2delta 4) were significantly downregulated in both male and female cKO brains, whereas *Akr1c1* (aldo‐keto reductase family 1 member C1) was upregulated (Figure 5b–d). Intriguingly, although the *Mkk7* gene was not deleted in oligodendrocytes (Figure 3), we did find alterations to the expression of oligodendrocyte genes related to myelination. mRNA levels of *Opalin* (oligodendrocytic myelin paranodal and inner loop protein), which is a central nervous system‐specific myelin protein, were profoundly decreased in male and female cKO brains (Figure 5e), whereas mRNA levels of *Enpp6* (ectonucleotide pyrophosphatase/phosphodiesterase 6), which is a choline‐specific glycerophosphodiesterase, and *Klk6* (kallikrein‐related peptidase 6), which is a serine protease, were dramatically increased (Figure 5f, g). These results indicate that MKK7 regulates neural functions and also affects oligodendrocyte maturation.

## 3 DISCUSSION

Genetically engineered mouse models have provided a broad range of insights into parental behavior (Kuroda et al., 2011). Various groups have reported that ablation of genes involved in signaling pathways (*Adcy3, Cin85, Creb1, Dbh, Eph5* and *FosB*), regulation of gene expression (*Hp1bp3* and *Peg1*) or cytoskeletal structure (*Mtap6*) results in abnormal nest building and failed pup retrieval (Table S3). Mice deficient for *Gabrd* ( γ‐aminobutyric acid receptor type A‐δ subunit) or *Tdag51* (T cell death‐associated gene 51) exhibit depression‐like symptoms after parturition as well as abnormal parental behavior (Maguire & Mody, 2008; Mihalek et al., 1999; Yun et al., 2019). These mutant mouse studies suggest that depression is closely related to the impaired parental behavior. To our knowledge, ours is the first report to document the involvement of the MKK7‐JNK signaling pathway in the nest building and pup retrieval aspects of mouse parenting behavior, as well as in depression‐like behavior.

Our results show that the impaired JNK activity caused by loss of *Mkk7* in mature neurons exacerbates depression‐like behavior but not anxiety‐like behavior (Figures 2 and 4). However, our results stand in contrast to a previous report in which knockout of *Jnk1* in mice both alleviated depression‐like and anxiety‐like behaviors and increased neurogenesis in the hippocampus (Mohammad et al., 2018). One explanation of this discrepancy may lie in the fact that three JNK isozymes with overlapping functions and different localizations exist: JNK1, 2 and 3 (Brecht et al., 2005; Coffey et al., 2002; Lee et al., 1999). The activities of all three JNK isozymes are impaired in our cKO mice, whereas JNK2 and JNK3 are still active in *Jnk1^‐/‐^* mice. Another explanation may rely on the different cell types experiencing gene deletion. In our study, *Mkk7* was removed in mature neurons only. In contrast, Mohammed *et al*. deleted the *Jnk1* gene in whole animal (Mohammad et al., 2018). Thus, additional studies with even more specific knockout mice are needed to understand precisely how MKK7‐JNK signaling supports neural functions.

Our cDNA microarray analysis of brains of 8‐week‐old cKO mice revealed clear alterations in gene expression patterns that were dependent on *Mkk7* deletion (Figure 4). We note that, in our previous study (Yamasaki et al., 2017), there was no difference in gene expression patterns between control and cKO mice at 8 months of age. It is well known that aging and changing social environments affect gene expression in multiple tissues, including the brain (Jiang et al., 2001; Shavlakadze et al., 2019; Tung & Gilad, 2013). Thus, it may be preferable to examine gene‐specific effects in younger mice in the context of the nervous system.

Calcium is a prominent regulator of neural functions. Accordingly, alterations to voltage‐gated calcium channel genes, including *CACNA2D4*, have been implicated in human psychiatric and neurological disorders (Heyes et al., 2015; Van Den Bossche et al., 2012). Some ion channels, including GABA receptors, are also related to the regulation of mental conditions (Table S3). AKR1C1 (20α‐hydroxysteroid dehydrogenase) negatively regulates GABA_A_ by producing inactive metabolites of progesterone such as 20α‐hydroxy‐5α‐pregnan‐3‐one and 5α‐pregnane‐3α,20α‐diol (Herd et al., 2007; Penning et al., 2000; Puia et al., 1990). Progesterone metabolism is involved in the synthesis of neurosteroids, which alter neural functions through interactions with cell membrane receptors in the brain. The proper regulation of this pathway is important for normal brain function. Abnormal neurosteroid metabolism has been linked to pathological conditions associated with dysregulated neuronal inhibition, including pathological depression and anxiety (Belelli et al., 2006). It was also previously reported that *Creb1* knockout in mice leads to a defect in pup retrieval (Jin et al., 2005). In addition, adrenergic signaling has been implicated in parental behavior in rats and mice (Rosenberg et al., 1977; Thomas & Palmiter, 1997). Our study revealed that loss of *Mkk7* in mature neurons altered mRNA levels of genes governing the regulation of ion channels and cAMP signaling. Accordingly, we conclude that the disruption of these functions may be partly responsible for the impaired parental behavior of cKO mice.

Three oligodendrocyte genes related to myelination, namely *Opalin*, *Enpp6* and *Klk6* (Aruga et al., 2007; Morita et al., 2016; Yoshikawa et al., 2016), showed altered expression patterns in mature neurons lacking *Mkk7* (Figure 5e–g). A previous report has shown that neuronal activity promotes oligodendrogenesis and adaptive myelination in the brain (Gibson et al., 2014). Optogenetic stimulation of cortical layer V projection neurons was found to induce proliferation of oligodendrocyte precursor cells, to increase numbers of newly generated oligodendrocytes, and to thicken the myelin sheath. We therefore speculate that our *Mkk7*‐deficient mice may have impaired neural activity, resulting in abnormal gene expression in oligodendrocytes.

In conclusion, our study establishes that MKK7‐JNK signaling in mature neurons plays an important role in mammalian social behavior. Lack of this signaling impairs gene expression in neurons and oligodendrocytes, leading to depression‐like mental status and failed parental behavior. Future work will elucidate the precise mechanisms underlying these critical structural and functional aspects of neuronal biology.

## 4 EXPERIMENTAL PROCEDURES

### 4.1 Mice

Mice homozygous for the targeted (floxed) *Mkk7* allele were previously described (Schramek et al., 2011), as were neuron‐specific Cre deleter strain *Synapsin‐Cre* (*Syn‐Cre*) transgenic mice on a C57BL/6 background (Zhu et al., 2001). The generation of *Mkk7^flox/flox^Syn‐Cre* mice was described in our previous report (Yamasaki et al., 2017). *ROSA‐tdTomato* mice on the C57BL/6 background [B6.Cg‐Gt(ROSA)26Sortm9(CAG‐tdTomato)Hze/J] were originally obtained from the Jackson laboratory (Madisen et al., 2010) and maintained at the Mouse Facility of Tokyo Medical and Dental University. *ROSA‐tdTomato* mice were crossed with *Syn‐Cre* mice to generate progeny suitable for immunofluorescent staining analyses. All mice were housed in conventional cages in a room on a normal 12 hr light/dark schedule, with access to food ad libitum. Genotyping was performed by PCR on DNA isolated from ear punch biopsies using the oligonucleotide primers listed in Table S4. All the procedures conformed to guidelines established by the Institutional Animal Care and Use Committee of Tokyo Medical and Dental University. All animal experiments were approved by the Institutional Animal Care and Use Committee of Tokyo Medical and Dental University.

### 4.2 Pup survival and retrieval

To evaluate parental behavior toward pups, separate cages were set up so that each contained a single pregnant control or *Mkk7* cKO female mouse (14–21 weeks old; *n* = 8 or 7/each group). After parturition, the number of surviving offspring was counted on days P0, P1 and P10 after birth.

Pup retrieval assays were performed as described previously (Sairenji et al., 2017). Briefly, virgin control and cKO female mice (14–16 weeks old) were moved from their home cage to a new cage for 10 min. Three newborn pups were then placed in three corners of the home cage. When a virgin mouse was returned to the center of her home cage, the test was started. The efforts of each virgin mouse to retrieve the 3 pups and bring them to the nest were monitored over a 1,200 s observation period. If no pups were retrieved within 1,200 s, the retrieval time was deemed to be 1,200 s. If the virgin mouse attacked the pups, the experiment was ended.

### 4.3 Open field test

Open field tests were performed as described previously (Aida et al., 2015). Briefly, a single control or cKO male mouse (*n* = 14 or 16/each group) was placed in an “open field” container (500 × 500 × 400 mm, O’Hara & Co.) for 30 min and videotaped. Time spent in the center of the container was recorded automatically using Image OF software (O’Hara) and expressed as a percentage of total time spent in the container.

### 4.4 Y‐maze test

The Y‐maze test was used to assess working memory in mice. The gray‐painted Y‐shaped maze had three arms of 400 mm in length, 150 mm in height and 30 mm in width, set at an angle of 120°. The test was conducted as described previously (Ishii et al., 2015; Maurice et al., 1994; Sarter et al., 1988). Briefly, a male mouse was placed at the end of the bottom arm and allowed to explore freely for 8 min. A series of arm entries chosen by the mouse over 8 min was recorded both visually and by video recording. When a mouse placed its hindpaws in an arm, the mouse was considered to have entered that arm. Alternation among arms entered spontaneously was defined as complete entry into each arm on overlapping triplet sets. The percentage alternation was calculated as the ratio of the actual (total alterations) to possible (total entries − 2) × 100.

### 4.5 Light–dark test

The light–dark box test was performed as described previously (Aida et al., 2015). The test apparatus (O’Hara) consisted of a cage (210 × 420 × 250 mm) divided into two sections of equal size by a partition containing a door. One section was the light chamber, which was brightly illuminated (390 lx). The other section was the dark chamber (2 lx). A male mouse was placed into the dark chamber and allowed to move freely between the two chambers via the open door for 10 min. The latency period before a mouse entered the light chamber was recorded automatically using Image LD software (O’Hara).

### 4.6 Forced swim test

The forced swim test was conducted using previously described methods (Porsolt et al., 1979; Su et al., 2013; Taylor et al., 2009). A male or female mouse was placed in a cylinder (250 mm tall, 130 mm diameter) filled with 15 mm of 25–27ºC water and forced to swim to survive. On day one, mice underwent the first session that lasted 15 min and was conducted without behavioral recording. On the following day, mice underwent the second session, which lasted 6 min and was conducted with behavioral recording. Immobility was quantified in the last 4 min of the test using EthoVision XT 7.0 software (Noldus).

### 4.7 Tail suspension test

The tail suspension test was performed as described previously (Cui et al., 2014). A male mouse was suspended humanely by the tail and attached to a bar 300 mm above the floor. Movement was monitored for 6 min using a charge‐coupled device camera, and duration of immobility was scored and analyzed using ImageJ TS software (O'Hara & Co.).

### 4.8 RNA preparation and cDNA microarray hybridization

Total RNAs were extracted from mouse brains using TRIzol reagent (15596018; Thermo Fisher Scientific, Inc.) and further purified using RNeasy Mini Kit (QIAGEN). The quality of RNA was initially assessed on a 1.5% agarose gel, and then determined by absorption spectrophotometry (DS‐11, DeNovix). Microarray analysis was entrusted to Takara Bio Inc. (Japan). cDNAs were synthesized using the Low Input Quick Amp Labelling Kit, One‐Color (Agilent Technologies). Cy3‐labeled cRNA was synthesized via an in vitro transcription reaction using 100 ng total RNA and T7 RNA polymerase. Following fragmentation, cRNA was hybridized for 17 hr, 10 r.p.m., at 65°C on the SurePrint G3 Mouse GE v2 8x60K Microarray using the Gene Expression Hybridization Kit (Agilent Technologies). GeneChips were washed using the Gene Expression Wash Pack (Agilent Technologies) and scanned using the SureScan Microarray Scanner (G2600D) (Agilent Technologies). Microarray data were processed using Agilent Feature Extraction. The Hierarchical Clustering and Heat Map drawings were produced using TM4 MeV software, version.4.8.1 (Saeed et al., 2003). Microarray data sets for the current study are available in Table S5.

### 4.9 Quantitative real‐time PCR

Quantitative RT‐PCR was performed as described previously with a slight modification (Hirayama et al., 2019). Total RNAs were extracted using TRIzol reagent (Thermo Fisher Scientific, Inc.) according to the manufacturer's instructions. cDNAs were synthesized using Superscript III Reverse Transcriptase (1792257; Thermo Fisher Scientific, Inc.) and oligo (dT) primer. qRT‐PCR reactions were performed using SsoFast EvaGreen Supermix (1725201; Bio‐Rad Laboratories, Inc.) and the CFX96 Real‐Time System (Bio‐Rad Laboratories, Inc.). PCR primer sequences used are listed in Table S4.

### 4.10 Fluorescence microscopy and immunofluorescence

These experiments were performed as described previously with slight modifications(Ma et al., 2017). *tdTomato^flox/+^Syn‐Cre* mice were deeply anesthetized with isoflurane and transcardially perfused with 4% paraformaldehyde. Fixed tissues were removed and cryoprotected by overnight immersion in 30% sucrose, followed by embedding in OCT compound (4583; Sakura Fintek). Sections were cut to a thickness of 40 μm using Cryostats (CM5030s; Leica Microsystems) and floated in PBS. For immunostaining, tissue sections were permeabilized with 0.5% Triton X‐100 in PBS and incubated overnight with primary antibodies (Abs) that recognized: Sox2 (Y‐17) (sc‐17320; Santa Cruz Biotechnology), DCX (ab18723; Abcam), NeuN (MAB377; Sigma‐Aldrich) or Olig2 (ab109186; Abcam). Tissues were then incubated with anti‐rabbit (A32790; Thermo Fisher Scientific, Inc.), anti‐mouse (A32766; Thermo Fisher Scientific, Inc.) or anti‐goat (A11055; Thermo Fisher Scientific, Inc.) Ab conjugated to Alexa Fluor 488. Nuclei were detected by staining with 1 μg/ml Hoechst 33342 (H3570; Thermo Fisher Scientific, Inc.). Stained tissue sections were washed and mounted using Mowiol (475904; CALBIOCHEM). Confocal images were acquired with a Zeiss LSM 710 confocal microscope (Carl Zeiss) and analyzed by ZEN 2012 Black Edition (Carl Zeiss).

### 4.11 Statistical analyses

Statistical analyses were performed as described previously (Kofuji et al., 2019). Statistical significance (*p* < .05) was calculated by GraphPad Prism 8 (GraphPad Software). One‐way ANOVA with Tukey's multiple comparison test was used for the analysis of nurturing behavior. The two‐tailed unpaired *t* test was used for the analyses of pup retrieval assays, open field test, Y‐maze test, light–dark test, tail suspension test, forced swim test, quantitative RT‐PCR and cDNA microarray.

## Supporting information

Table S1‐S5

Video S1

Video S2

Supplementary Material
