# Supplementary material for: MKK7 deficiency in mature neurons impairs parental behavior in mice
Source: Genes Cells. 2020 Nov 18;26(1):5–17. doi: 10.1111/gtc.12816 (PMC7839552; doi:10.1111/gtc.12816)
Supplement: Supplementary file 4 — Supplementary Material [file GTC-26-5-s004.pdf]

Supplementary Material

Supplementary Method

Immunoblotting

Proteins were extracted from tissues in RIPA buffer [50 mM Tris-HCl (pH7.6), 150 mM NaCl, 10 mM EDTA, 1% Nonidet P-40, 0.5% sodium deoxycholate, 0.1% sodium dodecyl sulfate, 8.5 μg/ml aprotinin, 1 mM phenylmethylsulfonyl fluoride]. Protein concentrations were quantified using the PierceTM BCA Protein Assay Kit (23227; Thermo Fisher Scientific, Inc.). Immunoblotting was performed as follows. Protein extracts were fractionated by SDS-PAGE and transferred to a polyvinylidene difluoride (PVDF) membrane (IPVH00010; Millipore), which was incubated in Blocking One (0395-95; Nacalai Tesque, Inc.) for 1 h. The blocked membrane was incubated overnight in Can Get Signal Solution (NKB-101; TOYOBO) with antibodies recognizing the following proteins: phospho-JNK (9251), JNK (9252), phospho-c-Jun (3270), c-Jun (9165), β-tubulin (2128) (all from Cell Signaling Technology), MKK7 (SAB1404255; Sigma-Aldrich), or MKK4 (sc-837; Santa Cruz Biotechnology). The membrane was washed in 0.05% TBS-Tween 20, incubated for 2 h with anti-mouse (7076; Cell Signaling Technology) or anti-rabbit (7074; Cell Signaling Technology) horseradish peroxidase-linked secondary antibody, and washed three times in 0.05% TBS-Tween 20. Proteins were detected using ECL solution (1.25 mM Luminol, 200 μM p-comeric acid, 100 mM Tris pH8.6, 0.03% hydrogen peroxide) or SuperSignal West Femto Maximum Sensitivity Substrate (34096; Thermo Fisher Scientific, Inc.) and ChemiDoc XRS (Bio-Rad Laboratories, Inc.) or Amersham Imager 680 (GE Healthcare Life Sciences). Phosphorylation states of c-Jun and JunB were examined using Phos-tag SDS-PAGE, which is a phospho-affinity SDS-PAGE protocol developed by Kinoshita *et al.* (Kinoshita, Kinoshita-Kikuta, & Koike, 2009; Kinoshita, Kinoshita-Kikuta, Takimiya, & Koike, 2006). Briefly, protein samples were concentrated and purified by trichloroacetic acid (TCA) precipitation prior to fractionation on an 8% polyacrylamide gel containing 50 μM Phos-tag acrylamide (AAL-107; NARD Institute, Ltd.) plus 100 μM MnCl2. Immunoblotting was carried out as described above.

Supplementary Figure

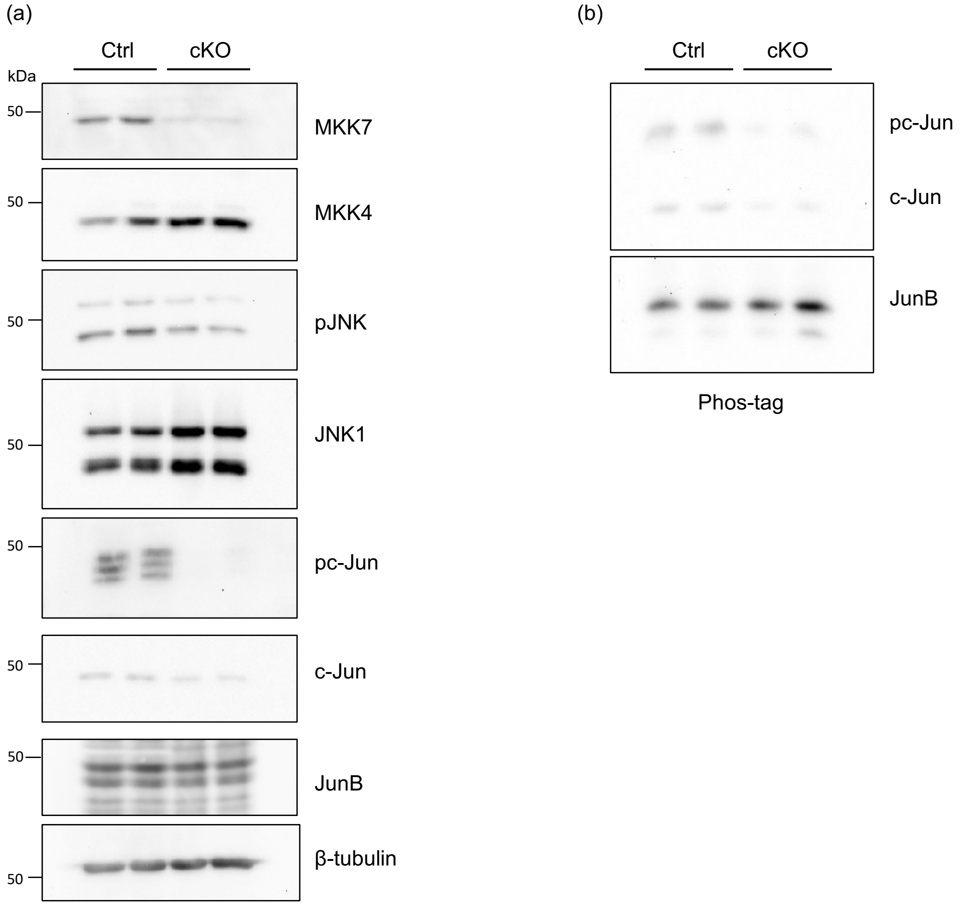

Fig. S1. Impaired activation of JNK signaling in *Mkk7*$^{flox/flox}$*Syn-Cre* mouse brains. Protein extracts of the brains of control and *Mkk7*$^{flox/flox}$*Syn-Cre* (cKO) mice at 10 weeks of age were subjected to immunoblotting by either standard SDS-PAGE (a) or Phos-tag SDS-PAGE (b) to detect the indicated proteins.

Video S1

Pup retrieval assay conducted using a 16-week-old control female virgin mouse. One P3 pup was placed in each of three corners of the home cage and the subject mouse's ability to bring them back to the nest was monitored. This about 5 minute video is shown here at 8x speed.

Video S2

Pup retrieval assay conducted using a 16-week-old cKO female virgin mouse. One P3 pup was placed in each of three corners the home cage and the subject mouse's ability to bring them back to the nest was monitored. This 20 minute video is shown here at 20x speed.

Supplementary References

Kinoshita, E., Kinoshita-Kikuta, E., & Koike, T. (2009). Separation and detection of large phosphoproteins using phos-tag sds-page. *Nature Protocols*, *4*(10), 1513–1521. https://doi.org/10.1038/nprot.2009.154

Kinoshita, E., Kinoshita-Kikuta, E., Takiyama, K., & Koike, T. (2006). Phosphate-binding tag, a new tool to visualize phosphorylated proteins. *Molecular and Cellular Proteomics*, *5*(4), 749–757. https://doi.org/10.1074/mcp.T500024-MCP200
